# The impact of operative approach on outcome of surgery for gastro-oesophageal tumours

**DOI:** 10.1186/1477-7819-5-95

**Published:** 2007-08-20

**Authors:** Stuart A Suttie, Alan GK Li, Martha Quinn, Kenneth GM Park

**Affiliations:** 1 Department of Surgery, Ward 33, Aberdeen Royal Infirmary, Foresterhill, Aberdeen, AB25 2ZN, UK

## Abstract

**Background:**

The choice of operation for tumours at or around the gastro-oesophageal junction remains controversial with little evidence to support one technique over another. This study examines the prevalence of margin involvement and nodal disease and their impact on outcome following three surgical approaches (Ivor Lewis, transhiatal and left thoraco-laparotomy) for these tumours.

**Methods:**

A retrospective analysis was conducted of patients undergoing surgery for distal oesophageal and gastro-oesophageal junction tumours by a single surgeon over ten years. Comparisons were undertaken in terms of tumour clearance, nodal yield, postoperative morbidity, mortality, and median survival. All patients were followed up until death or the end of the data collection (mean follow up 33.2 months).

**Results:**

A total of 104 patients were operated on of which 102 underwent resection (98%). Median age was 64.1 yrs (range 32.1–79.4) with 77 males and 25 females. Procedures included 29 Ivor Lewis, 31 transhiatal and 42 left-thoraco-laparotomies. Postoperative mortality was 2.9% and median survival 23 months. Margin involvement was 24.1% (two distal, one proximal and 17 circumferential margins). Operative approach had no significant effect on nodal clearance, margin involvement, postoperative mortality or morbidity and survival. Lymph node positive disease had a significantly worse median survival of 15.8 months compared to 39.7 months for node negative (*p *= 0.007), irrespective of approach.

**Conclusion:**

Surgical approach had no effect on postoperative mortality, circumferential tumour, nodal clearance or survival. This suggests that the choice of operative approach for tumours at the gastro-oesophageal junction may be based on the individual patient and tumour location rather than surgical dogma.

## Background

Distal oesophageal and gastro-oesophageal junction (GOJ) tumours now represent the commonest oesophageal tumour type in many western countries [1]. GOJ tumours are further classified as either lower third oesophageal with GOJ involvement (Siewert type I), true junctional (Siewert type II) or gastric cardia/fundal cancers with GOJ involvement (Siewert type III) [2]. In practice the precise classification is difficult and this may pose difficulties when deciding on the optimal surgical approach. Although a transabdominal technique is applicable to surgical resection of tumours of the gastric cardia/fundus (Siewert type III), a number of different approaches have been employed for surgical resection of cancer of the distal oesophagus and Siewert type I and II GOJ tumours.

It is claimed that the surgical approach used for these tumours may influence the ability to obtain tumour clearance and therefore impact upon survival. However, studies directly comparing different surgical approaches are difficult to interpret and have yielded contradictory results. The Ivor Lewis transthoracic and transhiatal approaches have been compared in patients with oesophageal cancer in terms of duration of procedure, hospital stay, postoperative outcome and survival, with no obvious benefit to either approach [3-8]. These studies include three randomised controlled trials and show no significant difference in rates of anastomotic leakage, postoperative mortality or survival between the approaches [4-6]. Only three studies have addressed specifically tumours of the distal oesophagus, GOJ and gastric cardia, with only Sasako *et al*., noting a higher morbidity in patients undergoing the left thoraco-laparotomy approach in comparison to transhiatal techniques [8-10]. Population based figures from the Scottish Audit of Gastric and Oesophageal Cancer (SAGOC), showed that there was little difference in outcome between the three commonest operative approaches for oesophageal cancer i.e. transhiatal, Ivor Lewis and left thoraco-laparotomy [11].

Irrespective of the approach utilised, positive surgical resection margins have been shown to adversely impact upon loco-regional recurrence and long term survival in oesophageal cancer patients [12-15]. Although achieving adequate nodal clearance *per se *has not been shown to the influence prognosis, the lymph node yield carries prognostic information in the presence of node positive disease [4,7,16].

This study focuses on tumours of the distal oesophagus and the gastro-oesophageal junction (Siewert types I and II) which now represent the commonest tumour type in western societies. It compares three surgical approaches in terms of resection margin clearance, lymph node yield and the prevalence of positive nodal disease and their impact on outcome.

## Methods

Data was collected prospectively and analysed retrospectively on patients with oesophageal and gastro-oesophageal (GOJ) tumours undergoing potentially curative surgery between 1994 and 2003.

### Tumour location

Analysis was focused on oesophageal tumours in the distal third of the oesophagus (>33 cm *ab orum*) and type I and II tumours of the GOJ according to the Siewert classification [2]:

Type I tumours – adenocarcinoma of the distal oesophagus with the bulk of the disease 1 to 5 cm above the GOJ, arising from Barrett's epithelium

Type II tumours – true adenocarcinoma of the cardia arising from the cardiac epithelium or short segments with intestinal metaplasia at the GOJ, with the bulk of tumour 1 cm above to 2 cm below the GOJ.

### Clinical staging

All subjects were medically fit (ASA grade I – III, WHO performance status ≤ 2) and underwent initial staging consisting of endoscopy, chest radiograph and thoracic and abdominal computerised tomography (CT) scan with contrast. Abdominal ultrasound scanning was performed to evaluate any abnormalities identified on the abdominal CT and a barium swallow performed if the endoscope was unable to traverse the lesion. During the final year of the study patients underwent endoscopic ultrasound as part of the staging process. Staging laparoscopy was performed to assess some type I and II tumours, based on radiological findings and at the surgeons discretion. All patients were discussed within the Upper Gastro-intestinal Multi-Disciplinary Team meeting, consisting of oncologists, radiologists and surgeons with a sub-specialty interest in gastro-oesophageal disease.

### Surgery

Surgery was performed two to four weeks following the completion of neo-adjuvant chemotherapy. During the early study period, patients were randomised to receive neo-adjuvant or no neo-adjuvant chemotherapy as part of the OE02 trial, while latterly all patients received neo-adjuvant chemotherapy based on the results of this trial [17]. All but seven patients completed the two cycles of chemotherapy.

One of three surgical approaches (Ivor Lewis, transhiatal or left thoraco-laparotomy) was performed by a single surgeon at a single institution. A gastric tube was formed for the neo-oesophagus for all patients. A left thoraco-laparotomy approach through the 8^th ^intercostal space was the operation of choice for GOJ tumours in the early part of the study. This was gradually phased out and replaced by a transhiatal approach during the study period and is now generally reserved for patients with deep chests or who are obese.

The transhiatal approach was used selectively for tumours less than 6 cm in length and when the whole tumour could be dissected under direct vision from within the abdomen after enlargement of the hiatus with a left sided cervical anastomosis. Two patients were converted from the transhiatal to Ivor Lewis approach during surgery.

### Pathology

Pathological staging was performed according to the criteria from the American Joint Committee on Cancer [18]. All tumours underwent complete macroscopic clearance (R_0_/R_1_). Overall margin involvement included, either distal, proximal and/or circumferential resection margins (CRM). Evidence of tumour 1 mm or less from any surface was taken as a positive margin. The lymph node yield as well as the number of tumour positive lymph nodes was documented.

### Follow up

All patients were followed up until death or the end of the data collection (August 2004) with a mean follow up of 33.2 months. Documented postoperative morbidity included anastomotic leak (based on clinical and/or radiological evidence with all patients undergoing a water soluble contrast swallow on the fifth post operative day), chest infection/pneumonia (clinical and/or radiological evidence) and cardiac complications (myocardial infarction/ischaemia or dysrythmias on electrocardiogram or elevation in cardiac enzymes). Postoperative mortality was defined as 30-day mortality. In hospital mortality is also depicted.

### Statistical analysis

Survival analysis was performed using Kaplan-Meier survival curves with comparisons drawn using log rank test. Test of association used the Chi-squared statistic, Fishers exact test (2-sided) or One Way ANOVA (Statistical Package for Social Sciences (SPSS) version 12.1). Statistical significance was denoted by a *p *value of <0.05.

## Results

### Patient and tumour characteristics

104 patients with distal third oesophageal or type I/II GOJ tumours underwent surgery within a 10 year period. Surgical resection was possible in 102 patients (98%). In two patients abdominal exploration identified more advanced disease than had been evident on preoperative staging precluding resectional surgery. There was no significant difference in patient demographics between each of the operative approaches used (table 1) with a median age of 64.1 years (range 32.1 – 79.4), with 77 males and 25 females. As expected, adenocarcinoma was the dominant histological type with tumours at this location.

**Table 1:** Patient and tumour characteristics

Characteristic		Ivor Lewis	Transhiatal	Left Thoraco-laparotomy	*p *value
Number Resected		29	31	42	
Median Age (years)		61.8	64.0	66.6	0.858
Male		20	23	34	0.503
Histology	Adenocarcinoma	22	29	37	0.125
	Squamous Cell Carcinoma	7	2	5	
Stage	I	5	6	0	0.013
	IIA	13	10	17	0.594
	IIB	2	7	5	0.191
	III	8	8	20	0.092
	IVA	1	0	0	0.281
Tumour Location	Oesophageal	21	12	13	0.002
	GOJ	8	19	29	
Neoadjuvant chemotherapy		11	15	17	0.686

### Tumour location and stage

46 (45%) tumours were located within the distal oesophagus whilst 56 tumours were classified as GOJ tumours. As expected, the Ivor Lewis approach was performed more often for distal third oesophageal tumours and transhiatal/left thoraco-laparotomy approach more often for GOJ tumours (*p *= 0.002) (table 1). There were significantly fewer stage I tumours within the left thoraco-laparotomy group (*p *= 0.013) although the proportion of stage I tumours was similar for both the Ivor Lewis and transhiatal groups.

### Treatment

**Figure 1:**
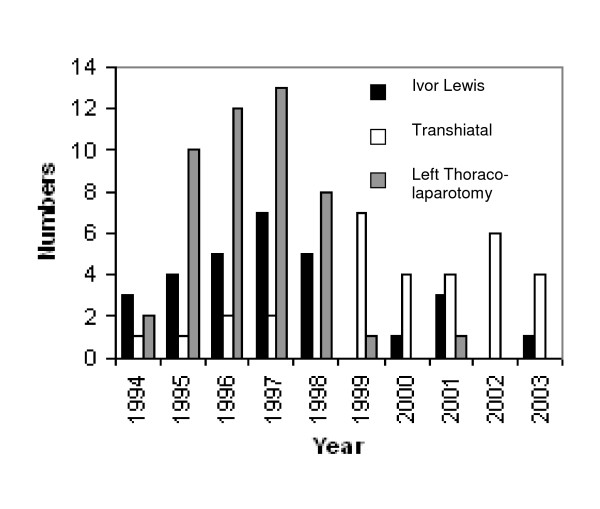
Distribution of procedures over time.

There was no difference in the proportion of patients receiving neo-adjuvant chemotherapy between each of the operative approaches used as all patients were subject to identical randomisation protocols for the OEO2 trial (table 1). Figure 1 displays the distribution of each procedure over the study period demonstrating a phased withdrawal of the thoraco-laparotomy approach in favour of a transhiatal technique.

### Postoperative morbidity and mortality

Overall 53 patients (52%) suffered a significant postoperative complication, the most common of which was chest infection (31%), followed by cardiac events (11%) and anastomotic leak (10%) (table 2). There was no statistically significant difference between the incidence of complications and the different surgical approaches (*p *= 0.864), although chest infections tended to occur more frequently in patients undergoing an Ivor Lewis approach and anastomotic leakage was more common in patients with a neck anastomosis in the transhiatal approach. The overall postoperative mortality was 2.9%, with similar rates for each of the three techniques used (table 3). In hospital mortality was 4.9%. Tumour location had no impact on postoperative mortality.

**Table 2:** Postoperative morbidity by approach

	Ivor Lewis n = 29	Transhiatal n = 31	Left Thoraco-laparotomy n = 42	*p *value
Chest Infection	12 (41%)	7 (23%)	13 (31%)	0.292
Cardiac Event	3 (10%)	2 (6%)	6 (14%)	0.564
Anastomotic Leak	1 (3%)	5 (16%)	4 (10%)	0.255

**Table 3:** Postoperative mortality and survival

	Procedure			*p *value	Tumour Location		*p *value
			
	Ivor Lewis n = 29	Transhiatal n = 31	Left Thoraco-laparotomy n = 42		Oesophageal n = 46	GOJ n = 56	
Postoperative Mortality	2	1	0	-	2	1	-
Median Survival (months)	18	44	17	0.395	33	22	0.530

### Survival

**Figure 2:**
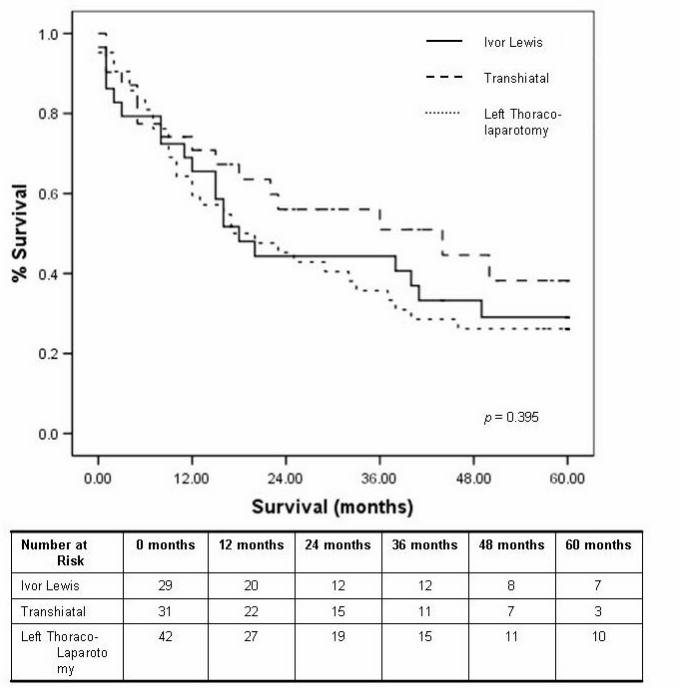
Kaplan Meier five year survival curves by procedure.

**Figure 3:**
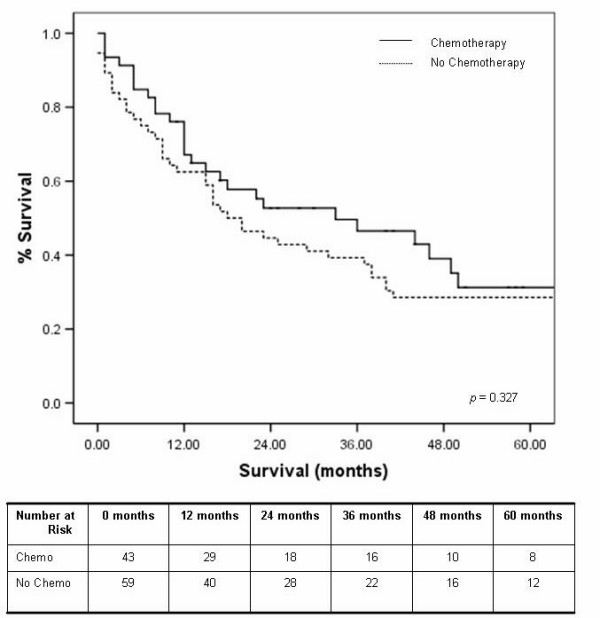
Kaplan Meier five year survival curves by neoadjuvant chemotherapy.

**Figure 4:**
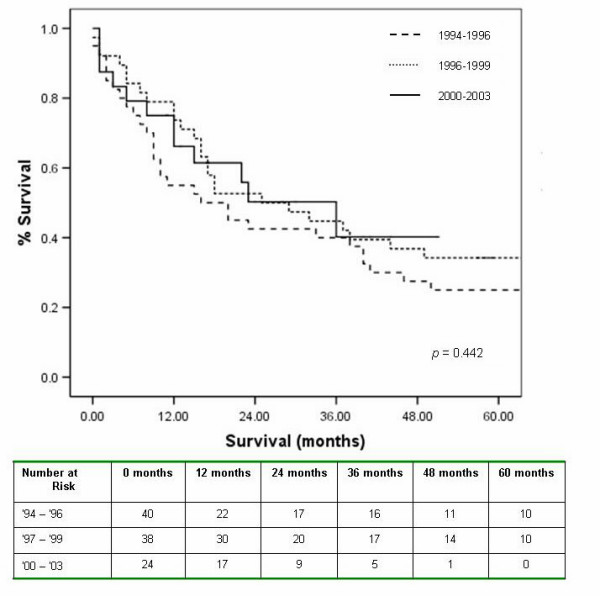
Influence of study period on five year survival.

Irrespective of the approach, the overall median survival was 23 months, with a one and five year survival of 68% and 20% respectively. The median survival for those undergoing the Ivor Lewis, transhiatal and left thoraco-laparotomy were 18, 44 and 17 months respectively (*p *= 0.395), with five year survival displayed in figure 2. Tumour location had no impact on survival (table 3). The use of neo-adjuvant chemotherapy had no significant effect on five year survival, (figure 3). The study period was analysed as an influential variable on survival and was found to have no significant impact on five year survival, *p *= 0.442 (figure 4).

### Lymph node yield

**Figure 5:**
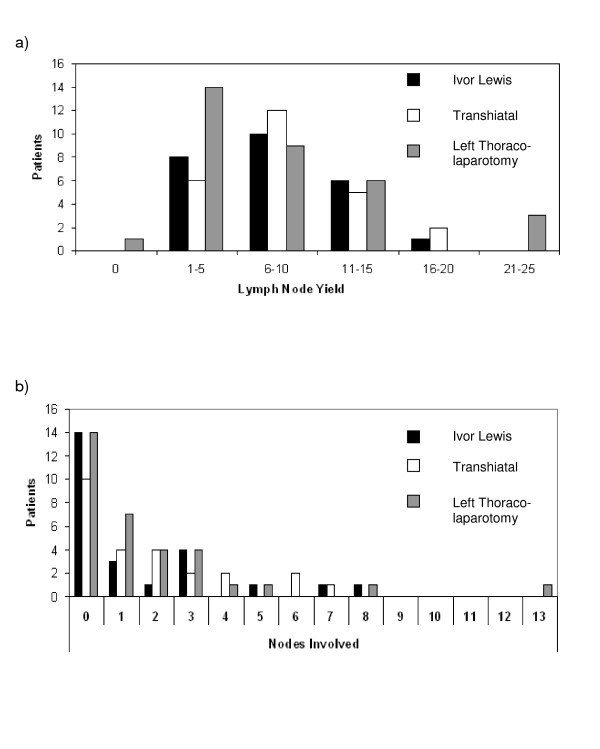
Lymph nodes 5a: Lymph node yield, 5b: Number of involved nodes by procedure.

**Figure 6:**
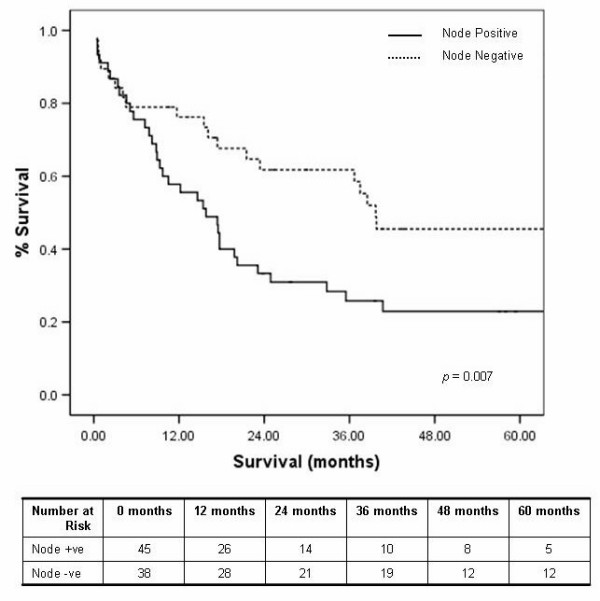
Kaplan Meier five year survival curves by nodal involvement.

Comparisons of nodal yield and tumour margins were based on 83 cases with full pathological data available (25 Ivor Lewis, 25 transhiatal and 33 left thoraco-laparotomy). The median number of resected nodes was similar irrespective of the operative approach used, Ivor Lewis (9, range 2–16), transhiatal (8, range 1–18) and left thoraco-laparotomy (7, range 0–23) (*p *= 0.285) (figure 5a). Tumour location had no effect on nodal yield (*p *= 0.898). In those with full pathology data available (n = 83), 45 patients had one or more lymph nodes positive for tumour. The median number of lymph nodes involved were 0 (range 0–8), 1 (range 0–7) and 1 (range 0–13) respectively for the Ivor Lewis, transhiatal and left thoraco-laparotomy approaches (figure 5b). Lymph node positive tumours had a significantly reduced median survival of 15.8 months in comparison to 39.7 months for lymph node negative tumours (*p *= 0.007) (figure 6).

### Circumferential tumour margin

Overall margin involvement, including two distal, one proximal and 17 circumferential margins (CRM), was 24.1% (20 of 83) with a positive CRM accounting for 85% of these cases. There was no significant difference between the three techniques in the prevalence of CRM involvement (Ivor Lewis 5, transhiatal 6, left thoraco-laparotomy 6) (*p *= 0.860) (table 4). Tumour location had no impact on CRM involvement (distal oesophageal 6, GOJ 11) (*p *= 0.451). Tumour T stage had a significant impact on CRM, *p *= 0.005, with all apart from one (T_2 _tumour, Ivor Lewis) CRM involvement occurring in T_3–4 _disease. Neo-adjuvant chemotherapy did not affect CRM involvement (*p *= 0.172).

**Table 4:** Margin involvement

Resection Margin		Procedure			*p *value
			
		Ivor Lewis	Transhiatal	Left Thoraco-laparotomy	
Overall	Margins Clear	19	18	26	0.836
	Margins Involved	6	7	7	
Margins Involved	Proximal	0	0	1	-
	Distal	1	1	0	-
	CRM	5	6	6	0.860

**Figure 7:**
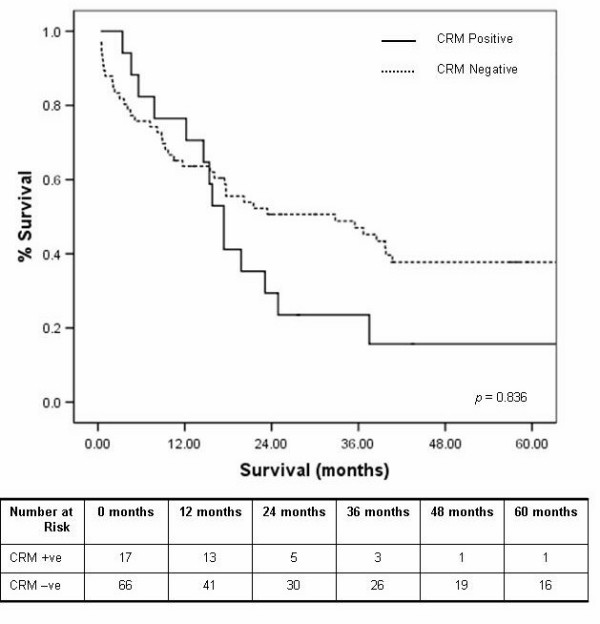
Kaplan Meier five year survival curves by circumferential resection margin involvement.

There was no significant difference in the median survival between overall (inclusive of circumferential, proximal and distal resection margins) positive and negative resection margins of 17.4 and 23.4 months, respectively, (*p *= 0.836) irrespective of surgical approach used. Although the difference in median survival of CRM positive patients was worse, at 17.4 months compared to 32.8 months for CRM negative tumours (figure 7), this did not reach statistical significance (*p *= 0.195). Higher T staging lead to a more likely positive CRM and therefore a trend effect on survival (although not significant). This is intuitive and as shown in our study in that all but one of the CRM positives occurred in higher T stage tumours. The one case of a T_2 _tumour with a positive CRM was clearly a disappointing reflection of inadequate surgical clearance.

## Discussion

The choice of operation for tumours at or around the gastro-oesophageal junction remains controversial with little evidence to support one technique over another. Indeed a population based audit demonstrated a number of different techniques used for oesophageal and gastro-oesophageal cancers, the three most common being: Ivor Lewis (30%), left thoraco-laparotomy (30%) and transhiatal (15%) [11].

In this study we have compared these three approaches undertaken by a single surgeon, over a 10 year period, so eliminating inter-operator variability, which may be greater than the differences between techniques *per se*, as shown in previous surgical studies [19]. The reduction in the number of resections performed each year is explained by the employment of a further two surgeons undertaking oesophago-gastric cancer resections. The resections performed by the other two surgeons were deliberately not included in order to reduce inter-operator variability. This was not a randomised trial but rather a pragmatic and practical approach to tailor the operative technique to the individual patient and tumour location and length. The transhiatal approach was limited to tumours in which dissection could be performed under direct vision to beyond the tumour and the Ivor Lewis technique applied to longer and more proximally situated tumours. During the study period, there was a deliberate and phased withdrawal of the left thoraco-laparotomy approach which was then reserved for patients with deeper chests or obesity. The selection of surgical approach was therefore performed on the basis of tumour length and location (i.e. Ivor Lewis versus transhiatal/Left thoraco-laparotmy), and not on the basis of stage.

The overall postoperative mortality in this series (2.9%) compares favourably to population based figures [11] and individual series comparing the three procedures [20-22]. Similarly, the one-year survival in this series was 68% with a five-year survival of 20% (median survival was 23 months) which is similar to reported series [13]. Although some reports have suggested a trend towards reduced long term survival with the transhiatal approach, no significant difference between the procedures in terms of disease free survival have been reported. Furthermore these studies have included patients with disease of the mid oesophagus for which the transhiatal approach may not be appropriate [5,9]. In contrast this series found a trend towards improved survival amongst the transhiatal resections. This is likely to represent the selective approach employed rather than any oncological advantage to the transhiatal method of oesophagectomy.

We found in this series, as have others, that patients with positive lymph nodes had a significantly worse prognosis than lymph node negative patients [4,7]. The value of extended lymphadenectomy in oesophageal cancer remains controversial [5,23,24]. In this series the surgical approach did not influence the number of lymph nodes recovered and an *en-bloc *resection of a junctional tumour is possible with each of the approaches used. Although the number of harvested nodes remains low in this series compared with others [5,7], three studies have also reported a similar low node harvest [9,15,25] with Stark *et al*., removing on average of 10.7 and 10.8 nodes for the transhiatal and Ivor Lewis approaches, respectively [9]. The lymph node yield is not only reflective of the surgery, but also of the pathological reporting systems. During the study period, no clear guidelines existed in the United Kingdom [26] as to the minimum number of nodes to be assessed, in comparison with other consensus groups [27], a possible explanation for the low lymph node yield. Furthermore, there may have been relative under staging due to the use of neo-adjuvant chemotherapy.

A postoperative complication occurred in 52% of patients in this series, similar to that of national figures [11]. Although there was a trend towards a reduced incidence of pulmonary complications with the transhiatal approach, this did not reach statistical significance. A trend towards an increased cervical anastomotic leak rate was noted in transhiatal resections consistent with findings noted by some [9,28] but not all studies [5,29].

Tumour clearance was similar with each approach used with a positive CRM being present in 20.5% of cases, with neo-adjuvant chemotherapy having no effect on CRM involvement. This was lower than reported in the SAGOC study with a 31% positive CRM [11]. As expected, tumour T stage had a significant effect on circumferential tumour clearance, with only one T_2 _tumour having a circumferential positive margin. This was a clearly disappointing reflection of inadequate surgical clearance. Distribution of tumour stage was only significantly skewed in stage I tumours and as expected, this had no effect on the overall proportion of positive resection margins. Reported rates of CRM involvement in the literature vary from 7–47% and as well as reflecting surgical technique, they may also vary according to the definition of positive resection margins [3,12-14,30]. Higher rates have been reported when a strict definition of any tumour within 1 mm of the margin is included. This was the definition used in this study and in the SAGOC report where positive CRM patients had a one year survival of 39% compared to 68% with a negative CRM [11]. Furthermore, in a study comparing gastrectomy to oesophagectomy for type II and III tumours, Ito *et al *demonstrated margin involvement to be an independent prognostic factor [15]. It is therefore important that clear resection margins are achieved.

In this series, surgical approach did not alter margin involvement which may be due to our selection process, although it may be influenced by the small numbers. The overall rate of positive CRM must be reduced and strategies for doing so may include improved patient staging and neoadjuvant therapy. The impact of endoscopic ultrasound in this situation is being investigated as part of a major ongoing trial [31]. However, it is likely that surgical philosophy may be equally if not more important with surgery being reserved for patients in whom an R_0 _resection is most likely rather.

## Conclusion

In this series, the surgical approach for distal oesophageal and oesophago-gastric tumour resection had no effect on postoperative mortality, survival, circumferential tumour clearance or nodal yield. We suggest that the choice of operative approach for tumours at or around the gastro-oesophageal junction may be based upon the individual patient and tumour location and length rather than surgical dogma.

## Competing interests

The author(s) declare that they have no competing interests.

## Authors' contributions

SAS: Data collection, analysis and its interpretation and manuscript construction and editing

AGKL: Supervision of data collection and analysis. Interpretation of data with significant contributions to study design and drafting of manuscript.

MQ: Data collection and analysis and drafting of manuscript.

KGMP: Conceived study and participated in its design. Co-ordinated all aspects of the study including drafting and critically editing the manuscript (senior editor).

All authors read the final manuscript and approved it for publication.
